# Analgesic Prescribing in Medically Complex Adult Outpatients Aged ≥40 Years: A Hierarchical Analysis of Comorbidity-Associated Patterns

**DOI:** 10.3390/jcm15208013

**Published:** 2026-10-16

**Authors:** Aymen Ali Alqurain, Abdulkarim Alshammari, Faten Alhomoud, Farah Kais Alhomoud, Abdulkhaliq J. Alsalman, Mohammad Kanan Alshammari, Hassan Al Gharash, Abdullah Jawad Alshawaf

**Affiliations:** 1 Department of Clinical Practice, Faculty of Pharmacy, Northern Border University, Rafha 91911, Saudi Arabia; abdulkarim.alshammari@nbu.edu.sa (A.A.); abdulkhaliq.alsalman@nbu.edu.sa (A.J.A.); 2 Department of Pharmacy Practice, College of Pharmacy, Imam Abdulrahman Bin Faisal University, Dammam 31441, Saudi Arabia; falhomoud@iau.edu.sa (F.A.); fkalhomoud@iau.edu.sa (F.K.A.); 3 Department of Pharmaceutical Care, Northern Borders Cluster, Rafha Centeral Hospital, Rafha 76321, Saudi Arabia; mokaalshammari@moh.gov.sa; 4 Emergency and Intensive Care Department, College of Nursing, Northern Border University, Arar 73213, Saudi Arabia; hassan.algharash@nbu.edu.sa; 5 Department of Respiratory Therapy, Mohammed Al-Mana College for Medical Sciences, Dammam 34222, Saudi Arabia; a.alshawaf@machs.edu.sa

**Keywords:** analgesic prescribing, multimorbidity, polypharmacy, outpatients, analgesic exposure, pharmacoepidemiology

## Abstract

**Background/Objectives**: Selecting appropriate analgesic therapy in patients with multimorbidity is increasingly challenging because clinicians must balance pain management against comorbidities, polypharmacy, and medication-related risks. However, evidence describing how analgesic prescribing patterns vary across patients with different multimorbidity profiles in routine outpatient practice remains limited. This retrospective cross-sectional study examined associations between multimorbidity and prescribing patterns across paracetamol, non-steroidal anti-inflammatory drugs (NSAIDs), and opioids, and identified clinical characteristics associated with different hierarchical analgesic exposure groups among adults aged ≥40 years in Saudi Arabia. **Methods**: Electronic health record data were analyzed for adults aged ≥40 years attending multiple clinics in the Eastern Region of Saudi Arabia between January 2020 and December 2021. Analgesic exposure at the index outpatient visit was classified using a hierarchical framework into mutually exclusive paracetamol-only, NSAID-priority, opioid-priority, or non-use groups. Multinomial logistic regression was performed to identify clinical characteristics associated with assignment to the hierarchical analgesic exposure groups. **Results**: Among 41,083 patients, 29,383 (71.5%) received at least one analgesic prescription at the index visit. The NSAID-priority group was the largest exposure category, followed by opioid-priority and paracetamol-only groups. Hierarchical analgesic prescribing patterns differed across patient clinical profiles, with prescribing observed across patients with cardiovascular, metabolic, gastrointestinal, respiratory, and neuropsychiatric disorders. In adjusted analyses, arthritis-related disease was associated with slightly higher odds of NSAID-priority assignment, whereas ischemic heart disease, gastritis-related disease, and respiratory disorders were associated with lower odds of NSAID-priority assignment. Constipation and urinary incontinence were associated with lower odds of opioid-priority assignment. **Conclusions**: Analgesic prescribing was common among medically complex adult outpatients aged ≥40 years, and hierarchical prescribing patterns differed across patient clinical and comorbidity profiles. The hierarchical framework provided a pragmatic approach for characterizing prescribing across major analgesic classes while accounting for concurrent exposure. Most disease-specific associations were attenuated after multivariable adjustment, and the remaining associations were generally modest in magnitude. Owing to the cross-sectional design and absence of detailed treatment-indication and pain-related information, these findings represent prescribing associations and should not be interpreted as evidence of causal prescribing decisions or treatment appropriateness.

## 1. Introduction

Pain is one of the leading causes of disability and healthcare utilization worldwide, affecting physical function, quality of life, and the management of chronic disease [1]. As populations age and the prevalence of multimorbidity continues to increase, the demand for effective therapy has grown substantially [1,2]. Chronic musculoskeletal pain commonly coexists with multiple chronic conditions and contributes substantially to treatment burden, healthcare utilization, and impaired quality of life [3,4]. Analgesics, including paracetamol, non-steroidal anti-inflammatory drugs (NSAIDs), and opioids, remain among the most frequently prescribed medications for managing acute and chronic pain across outpatient settings [1]. Despite their therapeutic benefits, each analgesic class carries a distinct safety profile and potential adverse effects, making analgesic therapy a critical component of individualized pharmacological care, particularly in medically complex patients [5].

Prescribing an appropriate analgesic therapy becomes considerably more challenging when patients have multiple chronic conditions. Many analgesics can influence cardiovascular, gastrointestinal, renal, or central nervous system outcomes, meaning that the same medication may be appropriate for one patient but potentially harmful for another [2,6,7]. At the same time, multimorbidity is frequently accompanied by polypharmacy, increasing the risk of drug–drug interactions, treatment burden, and adverse drug events [8,9]. As a result, analgesic prescribing often requires clinicians to balance expected pain relief against medication-related risks while considering the patient’s overall clinical profile rather than focusing on pain alone.

Previous studies have examined analgesic prescribing and utilization in outpatient populations, including potentially inappropriate NSAID use, opioid-related harm, and prescribing patterns within specific patient groups [1,5,10,11,12]. However, much of this literature has evaluated individual analgesic classes or specific clinical populations, providing less information on prescribing across multiple analgesic classes within the same medically complex outpatient cohort [5,10,11]. This distinction is relevant because multimorbidity and polypharmacy are common among patients requiring pain management and may complicate the interpretation of observed medication-use patterns [3,4,8,9].

To address this gap, the present study examined prescribing patterns across paracetamol, NSAIDs, and opioids among adults aged ≥40 years using a large real-world electronic health record (HER) dataset from Saudi Arabia. A hierarchical exposure framework was used as a pragmatic analytical approach to generate mutually exclusive groups for comparative analysis, while concurrent analgesic prescribing was characterized separately. The study further evaluated associations between demographic and clinical characteristics and hierarchical analgesic exposure-group assignment. This approach was intended to provide a broader characterization of analgesic prescribing in the context of multimorbidity rather than to infer the clinical reasoning underlying individual prescribing decisions. By providing a large-scale assessment of analgesic prescribing in routine outpatient practice, this study sought to identify prescribing patterns that may inform future prospective research evaluating the clinical significance and safety implications of analgesic use in medically complex populations.

## 2. Materials and Methods

### 2.1. Study Design and Study Population

This retrospective cross-sectional study analyzed routinely collected electronic health record (EHR) data obtained from adult outpatients aged ≥40 years attending multiple ambulatory care clinics in the Eastern Region of Saudi Arabia between January 2020 and December 2021. The study used the first eligible outpatient encounter for each patient during the study period as the index visit, from which analgesic exposure, demographic characteristics, medication burden, and clinical variables were determined unless otherwise specified.

The study included patients attending outpatient clinics across 18 clinical specialty categories within Qatif Central Hospital, including internal medicine, orthopedics, neurology, otorhinolaryngology, cardiology, dental services, and other medical specialties. Internal medicine-related services represented the largest component of the source population. Accordingly, this was a single-center study encompassing multiple clinical specialties.

Patients were eligible for inclusion if they were aged 40 years or older and had at least one documented outpatient visit during the study period. This age threshold was prespecified to focus the analysis on middle-aged and older adults, in whom multimorbidity and concurrent medication use become increasingly prevalent and are particularly relevant when evaluating analgesic prescribing patterns [9].

Patients were excluded if they were younger than 40 years, had inpatient admissions, presented to the emergency department, or had encounters directly associated with surgical procedures, including day surgery and postoperative follow-up visits. Routine outpatient encounters within specialties involving surgical practice, including orthopedics, otorhinolaryngology, gynecology, urology, and dental care, were retained when they involved outpatient assessment, follow-up, or medical/conservative management rather than care directly associated with a surgical procedure. Encounters occurring outside the study period were also excluded.

For patients with multiple eligible outpatient encounters, only the first eligible encounter was included to ensure independence of observations and to avoid overrepresentation of patients with frequent healthcare utilization.

### 2.2. Data Sources and Variable Definitions

Data were extracted from the EHR system using a standardized data extraction template and subsequently processed for analysis. The extracted dataset included demographic characteristics, outpatient diagnosis and comorbidities, recent laboratory results, and complete medication prescribing records available at the index outpatient visit.

Prescription records identified analgesic exposure documented at the index outpatient encounter but did not reliably distinguish newly initiated from continued therapy.

Data quality was evaluated through standardized cleaning procedures, including the removal of duplicate records, validation of plausible value ranges, consistency checks across related variables, and harmonization of coding formats. All records were de-identified before analysis to maintain patient confidentiality.

Comorbidities were primarily identified from clinical conditions documented in the EHR at the index outpatient visit; previous encounters were not reviewed, and no retrospective diagnostic look-back period was applied. To supplement potentially unreported long-term conditions, medication information available at the index visit was reviewed using the validated Rx-Risk index, which identifies chronic conditions from medication prescribing records [13,14]. Clinical conditions available in the dataset were reviewed and grouped by the research team into clinically meaningful categories for analysis; they were not identified using a prespecified International Statistical Classification of Diseases, tenth version (ICD-10) code-based extraction algorithm [15]. ICD-10 terminology was subsequently used to support standardization of disease classification, while overall comorbidity burden was quantified using the Charlson Comorbidity Index (CCI) [16]. The CCI was calculated by summing the established weighted scores for the included comorbid conditions together with the corresponding age component.

Pain-associated conditions were identified from clinical conditions documented at the index outpatient visit that were associated with pain, including migraine/headache, back pain, arthritis-related pain, and other recorded pain-associated conditions. These conditions were reviewed and grouped by the research team into clinically meaningful categories. This variable represented the presence of a documented pain-associated condition rather than a direct assessment of pain symptoms, severity, duration, or treatment indication and did not distinguish acute from chronic pain. Analgesic prescriptions were not used to define this variable.

The Rx-Risk approach was used only as a supplementary aid for identifying potentially unreported chronic conditions and was not the primary method of comorbidity ascertainment. To avoid circular classification, prescriptions for paracetamol, NSAIDs, and opioids, which constituted the study exposure outcome, were not used through the Rx-Risk approach to define pain-associated conditions or other variables directly related to analgesic exposure. Pain-associated conditions were identified from the clinical-condition information available in the dataset and grouped by the research team independently of analgesic prescribing. In addition, NSAID prescribing was not used to identify or classify arthritis-related disease.

Several clinical conditions were represented as composite categories based on the diagnostic information available in the EHR. Arthritis-related diseases included osteoarthritis, rheumatoid arthritis, and nonspecific arthritis diagnoses; respiratory disorders included asthma and chronic obstructive pulmonary disease; pain-associated conditions included recorded headache, back pain, and musculoskeletal pain diagnoses; and gastritis-related diseases included gastro-oesophageal reflux disease, dyspepsia, peptic ulcer disease, and gastritis-related diagnoses. Osteoporosis was identified using the available diagnostic and medication-based disease information. Where applicable, medication-based identification followed the Rx-Risk approach, and qualifying prescribed products could contribute to identification of the corresponding clinical condition. Because the historical EHR data did not consistently retain sufficient diagnostic granularity to reconstruct every individual component code retrospectively, these variables were analysed as the predefined grouped clinical conditions available in the study dataset.

Creatinine clearance (CrCl) was calculated using the Cockcroft–Gault equation based on age, body weight, sex, and serum creatinine concentration, including the standard correction factor for female patients [17]. Actual body weight recorded in the EHR was used directly in the calculation because height and body mass index were unavailable, precluding consistent calculation of ideal or adjusted body weight. Serum creatinine measurements were restricted to a ±30-day window around the index outpatient visit, and the measurement closest to the index visit within this window was used. CrCl was available for all participants. Laboratory values were defined as the most recent values available on or before the index outpatient visit to reflect the patient’s baseline physiological status at the time of prescribing.

Missing data were assessed for all variables included in the analyses. Among the 41,084 eligible records, one participant had missing sex information (1/41,084; 0.002%). No missing observations were identified for age, body weight, CrCl, CCI, number of prescribed medications (NPM), or the clinical variables included in the regression analyses. The participant with missing sex information was excluded using complete-case analysis, resulting in a final analytic sample of 41,083 participants. No imputation was performed.

Medication prescribing records included long-term medications, short-term therapies, and nutritional supplements prescribed at the index visit. All medications were classified according to the Anatomical Therapeutic Chemical (ATC) Classification system [18]. Medication burden was quantified as the total number of prescribed medications (NPM) recorded at the index outpatient visit.

### 2.3. Analgesic Selection Definition and Classification

Analgesic exposure was determined from prescribing records documented at the index outpatient visit. Analgesics were classified into three therapeutic groups according to the ATC classification system: paracetamol (ATC code N02BE01), non-steroidal anti-inflammatory drugs (NSAIDs; ATC code M01A), and opioids (ATC code N02A). Medication mapping was verified across all three analgesic classes, including paracetamol, NSAIDs, and opioids, with particular attention to codeine-containing preparations and fixed-dose combination products.

Analgesic exposure was defined according to prescriptions for paracetamol, NSAIDs, and opioids recorded at the index outpatient visit. The available prescription records did not reliably distinguish newly initiated treatment from continuation of previously prescribed therapy. Exposure classification was therefore based on prescribing recorded at the index encounter and should not be interpreted as treatment initiation. Analgesic prescriptions from previous encounters that were not recorded at the index visit, over-the-counter analgesic use, and treatment duration were not incorporated into the exposure definition.

To generate mutually exclusive outcome categories for multinomial regression while accounting for concurrent analgesic prescribing, patients were assigned to a single hierarchical exposure group according to the highest-ranking analgesic class prescribed at the index outpatient visit. Patients prescribed an opioid were assigned to the opioid-priority group regardless of concomitant NSAID or paracetamol use. Among patients who were not prescribed an opioid, those prescribed an NSAID, with or without paracetamol, were assigned to the NSAID-priority group. Patients prescribed paracetamol without an opioid or NSAID were assigned to the paracetamol-only group, whereas patients receiving none of these analgesics were classified as non-users and served as the reference group.

The hierarchical ordering (opioid > NSAID > paracetamol) was prespecified as a pragmatic analytical convention to generate mutually exclusive outcome categories in the presence of concurrent analgesic prescribing. The ordering prioritized opioid exposure, followed by NSAID exposure and then paracetamol exposure, for analytical classification when more than one analgesic class was prescribed; it was not intended to imply therapeutic superiority, prescribing appropriateness, or the sequence of clinical decision-making. Consequently, patients assigned to the opioid-priority or NSAID-priority groups may also have received lower-priority analgesic classes. Concurrent prescribing patterns were therefore examined separately using descriptive analyses, and multinomial regression estimates were interpreted as associations with hierarchical group assignment rather than exclusive exposure to an individual analgesic class.

Exposure classification was restricted to prescriptions issued at the index outpatient visit. Prescriptions recorded before or after the index outpatient visit were not considered when defining exposure categories. Information on analgesic dose, treatment duration, and administration frequency was not consistently available within the EHR system and was therefore not incorporated into the exposure definition.

### 2.4. Bias, Confounding and Sample Size

Residual confounding and confounding by indication cannot be excluded because several factors likely to influence analgesic prescribing were unavailable within the EHR system, including treatment indication, pain severity and duration, over-the-counter analgesic use, non-pharmacological pain management, medication adherence, clinician prescribing rationale, and patient preferences. The available data therefore cannot reconstruct the clinical reasoning underlying individual prescriptions, and the analysis should be interpreted as describing associations between measured clinical characteristics and observed prescribing patterns rather than determinants of analgesic prescribing.

Because analgesic prescribing and clinical characteristics were assessed concurrently at the index outpatient visit, the observed associations describe the coexistence of clinical characteristics and hierarchical analgesic exposure-group assignment rather than temporal relationships. Accordingly, the findings should not be interpreted as evidence that a clinical condition influenced analgesic prescribing, resulted from analgesic treatment, or reflected a medication-related adverse effect.

All eligible patients meeting the predefined inclusion criteria during the study period were included in the analysis; therefore, no formal sample size calculation was performed. The use of the complete eligible outpatient cohort was expected to provide adequate statistical precision for both descriptive analyses and multivariable modelling.

### 2.5. Statistical Analysis

Baseline patient characteristics were summarized using descriptive statistics. Continuous variables were presented as mean (standard deviation [SD]) or median (interquartile range [IQR]) according to their distribution, whereas categorical variables were presented as frequencies and percentages. For comparisons across the four hierarchical analgesic exposure groups, overall group differences were assessed using one-way analysis of variance (ANOVA) for normally distributed continuous variables, Kruskal–Wallis tests for non-normally distributed continuous variables, and chi-square tests for categorical variables, as appropriate. Corresponding effect sizes were reported using η^2^ for one-way ANOVA, ε^2^ for Kruskal–Wallis tests, and Cramer’s V for categorical variables.

The primary analysis used multinomial logistic regression analysis to identify demographic and clinical factors associated with hierarchical analgesic exposure-group assignment. This modelling approach was prespecified because the hierarchical exposure framework generated four nominal, mutually exclusive outcome categories (non-use, paracetamol-only, NSAID-priority, and opioid-priority prescribing). The non-user group served as the reference category. Adjusted odds ratios (ORs) with corresponding 95% confidence intervals (95% CI) were estimated for each exposure group. Because exposure classification was hierarchical, the adjusted ORs represent associations with assignment to the highest-ranking analgesic exposure category rather than exclusive use of an individual analgesic class. Furthermore, because prescribing and clinical characteristics were assessed concurrently at the index outpatient visit, the adjusted ORs were interpreted as measures of association rather than treatment effects, medication-related adverse outcomes, or prescribing appropriateness.

Covariates were selected a priori based on clinical relevance and evidence from the existing literature. Age, sex, body weight, CrCl, CCI, NPM, and clinic specialty were retained as a fixed core adjustment set. Each individual clinical condition was evaluated separately by adding that condition to the same core adjustment model; individual clinical conditions were therefore not entered simultaneously into a single multinomial model. No stepwise or other data-driven variable-selection procedure was applied. CCI was retained as a measure of overall comorbidity burden, while individual clinical conditions were examined separately to estimate their associations with hierarchical analgesic exposure-group assignment conditional on the core adjustment variables. Multicollinearity among the core adjustment variables was assessed using VIFs, which ranged from 1.004 to 2.365. Model fit was assessed using likelihood-ratio and goodness-of-fit tests.

The functional form of continuous covariates was assessed, and evidence of nonlinearity was identified for body weight. Sensitivity analyses incorporating linear and quadratic weight terms were therefore performed. Interaction terms were not prespecified or included. Multivariable analyses were conducted using complete cases; no missing data imputation was performed.

The non-user group served as the reference outcome category. Binary clinical-condition variables were coded as 0 = absent and 1 = present, with absence specified as the reference category so that the reported odds ratios represent the presence versus absence of the respective clinical condition.

To assess the robustness of findings to the hierarchical exposure classification, sensitivity analyses were conducted using non-mutually exclusive binary indicators of any paracetamol, any NSAID, and any opioid prescribing. Separate binary logistic regression models were fitted for each analgesic class. Consistent with the primary analytical approach, age, sex, body weight, CrCl, CCI, and NPM were included as adjustment covariates, with each clinical condition evaluated individually after adjustment for these covariates. Results are reported as adjusted odds ratios (ORs) with 95% confidence intervals.

To assess the potential influence of the analgesic non-user reference group, a sensitivity analysis was performed restricting the cohort to patients with a documented pain-associated condition at the index visit. The same hierarchical analgesic exposure classification and core adjustment set used in the primary multinomial analysis were applied to this restricted cohort.

As an additional sensitivity analysis, the disease-specific multinomial logistic regression models were repeated without the CCI to assess whether adjustment for the composite comorbidity measure materially influenced the estimated associations between individual clinical conditions and hierarchical analgesic exposure-group assignment. Each clinical condition was entered separately, with adjustment for age, sex, body weight, CrCl, NPM, and clinical specialty. Analgesic non-use remained the reference outcome category.

Exploratory subgroup analyses compared demographic and clinical characteristics between male and female analgesic users and between older adults (≥65 years) and middle-aged adults (40–64 years). For exploratory comparisons between two subgroups, continuous variables were compared using the independent-samples t test or Mann–Whitney U test, and categorical variables using the chi-square or Fisher exact test, as appropriate. Age- and sex-stratified analyses were conducted as exploratory descriptive analyses to characterize variation in the clinical composition of the cohort across demographic strata and were not prespecified as tests of effect modification.

Statistical significance was defined as a two-sided *p*-value < 0.05. All analyses were conducted using IBM SPSS Statistics, version 26 (IBM Corp., Armonk, NY, USA).

### 2.6. Ethics and Reporting Standards

Ethical approval was obtained from the Institutional Review Board (IRB) of Mohammed Al-Mana College for Medical Sciences (SR/RP/79, approved 17 February 2022) and Qatif Central Hospital (QCH-SRECO 19/2022, approved 8 June 2022). The requirement for informed consent was waived because of the retrospective nature of the study and the use of fully de-identified EHR data. The study was conducted in accordance with the principles of the Declaration of Helsinki and all applicable institutional and regulatory requirements.

The reporting of this study followed the Strengthening the Reporting of Observational Studies in Epidemiology (STROBE) standards for cross-sectional studies [19].

## 3. Results

A total of 41,083 adult outpatients aged ≥40 years were included in the analysis. The median age was 52 years (IQR 45–62), and 20,800 patients (50.6%) were female. Among the 41,083 patients in the final analytical cohort, 29,383 (71.5%) had at least one prescription for paracetamol, an NSAID, or an opioid recorded at the index outpatient encounter. This proportion reflects prescriptions recorded at the index visit and does not distinguish newly initiated from continued therapy. Analgesic prescribing varied across the clinical specialties represented in the source population. The proportion of patients with at least one assessed analgesic prescription recorded at the index visit was 37.5% in cardiology, 38.1% in dental services, 72.8% in internal medicine, 78.6% in neurology, and 81.3% in orthopedics (Supplementary Table S4).

Under the hierarchical exposure framework, 14,021 patients (34.1%) were assigned to the NSAID-priority group, 7719 (18.8%) to the opioid-priority group, and 7643 (18.6%) to the paracetamol-only group; the remaining 11,700 (28.5%) were classified as analgesic non-users (Table 1).

Patients assigned to the opioid-priority group had a higher mean body weight and CrCl and included a greater proportion of male patients compared with those not assigned to the opioid-priority group. Pain-associated conditions were common throughout the cohort, although osteoporosis and arthritis-related diseases were less frequent in the opioid-priority group. Several cardiovascular, metabolic, gastrointestinal, respiratory, and neuropsychiatric conditions also varied across the exposure groups and were generally least common among patients assigned to the opioid-priority group. Full demographic and clinical comparisons are presented in Table 1. Although several characteristics differed statistically across hierarchical exposure groups, the corresponding effect sizes were generally small, indicating that statistical significance did not necessarily reflect substantial between-group differences.

**Figure 1:**
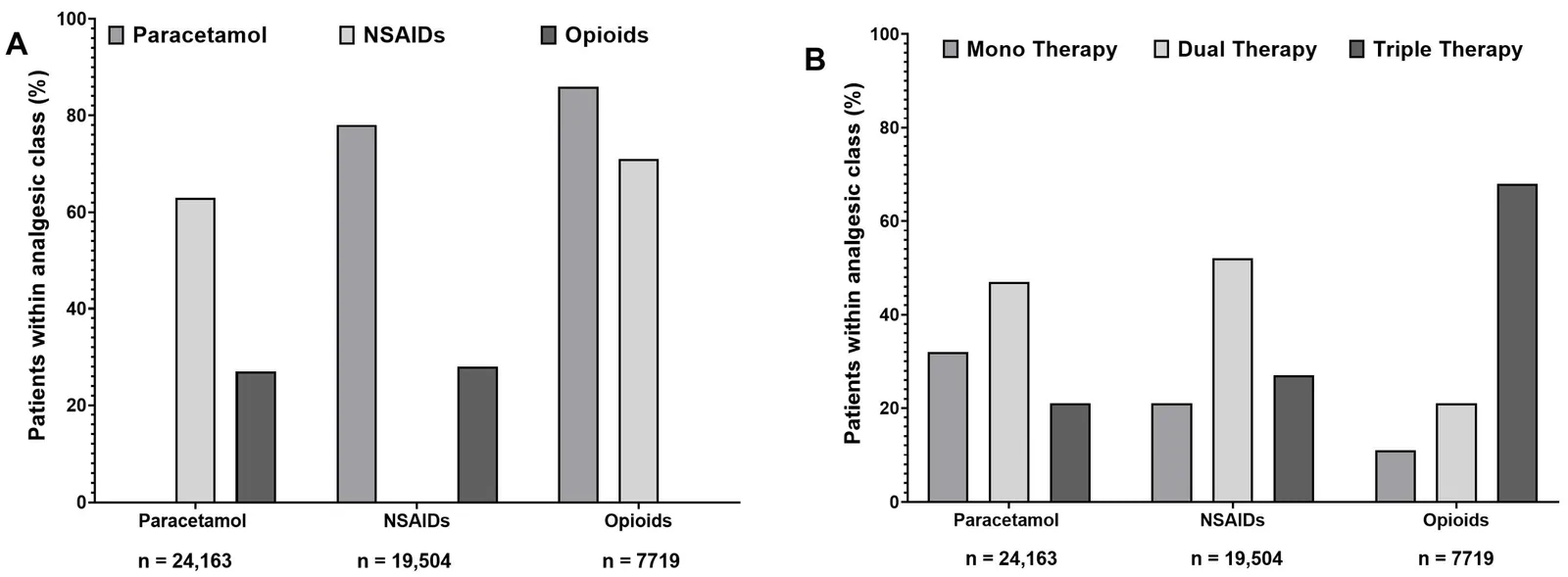
Concurrent analgesic prescribing patterns at the index outpatient visit. (**A**) Proportion of patients within each analgesic class who were concurrently prescribed either of the other analgesic classes. Among patients prescribed paracetamol (n = 24,163), 15,128 (62.6%) were also prescribed an NSAID and 6625 (27.4%) an opioid. Among NSAID users (n = 19,504), 15,128 (77.6%) were also prescribed paracetamol and 5483 (28.1%) an opioid. Among opioid users (n = 7719), 6625 (85.8%) were also prescribed paracetamol and 5483 (71.0%) an NSAID. (**B**) Distribution of monotherapy, dual-class therapy, and triple-class therapy within each analgesic class. For paracetamol, 7643 (31.6%) received paracetamol alone, 11,287 (46.7%) received dual-class therapy, and 5233 (21.7%) received all three classes. Corresponding values were 4126 (21.2%), 10,145 (52.0%), and 5233 (26.8%) among NSAID users, and 844 (10.9%), 1642 (21.3%), and 5233 (67.8%) among opioid users. NSAID; non-steroidal anti-inflammatory drug.

Because the hierarchical classification assigned patients according to the highest-priority analgesic class, concurrent prescribing patterns were examined separately. Among the 29,383 patients with at least one assessed analgesic prescription, 12,613 (42.9%) received one analgesic class, 11,537 (39.3%) received two classes, and 5233 (17.8%) received all three classes. In Figure 1A, the denominator for each analgesic class is the total number of patients prescribed that class (paracetamol, n = 24,163; NSAIDs, n = 19,504; opioids, n = 7719), and patients prescribed multiple classes could contribute to more than one concurrent-exposure category. The most frequent combination was paracetamol with an NSAID, while a substantial proportion of patients assigned to the opioid group also received paracetamol and/or an NSAID. Figure 1B presents monotherapy, dual-class therapy, and triple-class therapy patterns within each indexed analgesic class. Dual-class therapy predominated across most combinations, whereas triple-class prescribing was concentrated among patients receiving opioids.

Among analgesic users, female patients demonstrated a higher prevalence of osteoporosis, arthritis-related diseases, pain-associated conditions, gastritis-related diseases, depression, epilepsy, and respiratory disorders compared with male patients, whereas male patients had higher body weight and creatinine clearance values (Supplementary Materials Table S1).

Older adults had a greater prevalence of several cardiovascular and metabolic conditions, including heart failure, hypertension, ischemic heart disease, and diabetes mellitus. Mean creatinine clearance was slightly lower among older adults (93.93 ± 46.22 mL/min) than among middle-aged adults (99.37 ± 40.28 mL/min). In contrast, arthritis-related diseases and pain-associated conditions were proportionally more common among middle-aged individuals (Supplementary Materials Table S2).

**Figure 2:**
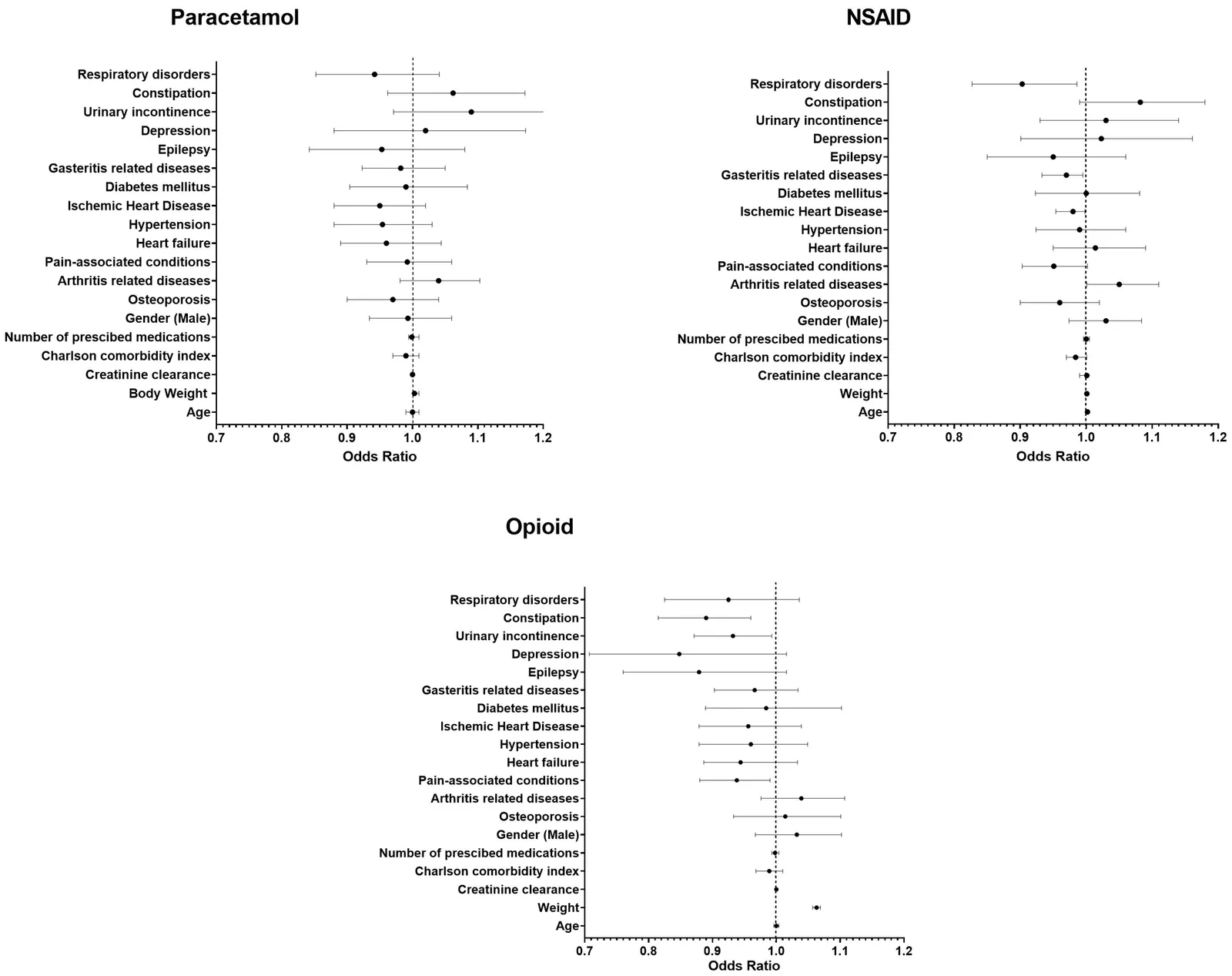
Multinomial logistic regression analysis of factors associated with hierarchical analgesic exposure-group assignment compared with analgesic non-use. CCI: Charlson comorbidity index, CrCl: Creatinine clearance, NPM: Number of prescribed medications. Adjusted odds ratios for assignment to the paracetamol-only, NSAID-priority, and opioid-priority groups relative to the analgesic non-user group are presented. Each clinical condition was evaluated in a separate multinomial regression model adjusted for age, sex, body weight, CrCl, CCI, NPM, and clinical specialty. Complete regression estimates are presented in Supplementary Table S3.

Multivariable multinomial logistic regression analysis demonstrated that several demographic and clinical characteristics were associated with hierarchical analgesic exposure-group assignment (Figure 2). Assessment of continuous-variable functional form indicated evidence of nonlinearity for body weight. In sensitivity analyses incorporating a quadratic weight term, the principal clinical associations observed in the primary models were preserved.

In the primary multinomial logistic regression analysis, adjusted for age, sex, body weight, CrCl, CCI, NPM, and clinical specialty, most crude disease-specific associations were substantially attenuated after adjustment (Table S3). Figure 2 demonstrated that arthritis-related disease was associated with slightly higher odds of NSAID-priority assignment (OR = 1.05, 95% CI 1.00–1.11). Ischemic heart disease and gastritis-related disease were associated with slightly lower odds of NSAID-priority assignment (OR = 0.97, 95% CI 0.95–0.99, and OR = 0.97, 95% CI 0.94–0.99, respectively). Respiratory disorders were similarly associated with lower odds of NSAID-priority assignment (OR = 0.90, 95% CI 0.83–0.99).

Urinary incontinence and constipation were associated with lower odds of opioid-priority assignment (OR = 0.93, 95% CI 0.87–0.99 and OR = 0.89, 95% CI 0.82–0.96, respectively). Most other adjusted disease-specific estimates included the null value. Among the core covariates, higher body weight was most clearly associated with opioid-priority assignment (OR = 1.06 per unit increase, 95% CI 1.06–1.07) (Figure 2).

In the non-mutually exclusive class-specific sensitivity analysis, in which patients could contribute to more than one analgesic class, several associations remained after multivariable adjustment (Table S5). Male sex was associated with lower odds of any paracetamol prescribing (OR = 0.93, 95% CI 0.90–0.97) and any opioid prescribing (OR = 0.89, 95% CI 0.84–0.94), while increasing body weight was associated with higher odds of both any paracetamol and any opioid prescribing, particularly opioid prescribing (OR = 1.01, 95% CI 1.01–1.02). Heart failure and hypertension were associated with slightly lower odds of any paracetamol prescribing, as were ischemic heart disease and gastritis-related disease. Respiratory disorders were associated with lower odds of any paracetamol (OR = 0.89, 95% CI 0.83–0.96) and any NSAID prescribing (OR = 0.91, 95% CI 0.85–0.98). Epilepsy and depression were associated with lower odds of any opioid prescribing (OR = 0.87, 95% CI 0.77–0.99 and OR = 0.77, 95% CI 0.66–0.90, respectively), while constipation showed a modest inverse association with any opioid prescribing (OR = 0.91, 95% CI 0.85–0.99). Overall, adjustment markedly attenuated many of the crude class-specific associations.

When the hierarchical analysis was restricted to patients with documented pain-associated conditions (n = 25,257), most disease-specific associations were similarly attenuated after adjustment for age, sex, body weight, CrCl, CCI, NPM, and clinical specialty (Table S6). Arthritis-related disease remained associated with slightly higher odds of NSAID-priority assignment (OR = 1.08, 95% CI 1.01–1.15), whereas gastritis-related disease was associated with slightly lower odds of NSAID-priority assignment (OR = 0.96, 95% CI 0.92–0.99). Urinary incontinence and constipation were associated with modestly lower odds of opioid-priority assignment (OR = 0.94, 95% CI 0.89–0.99 and OR = 0.93, 95% CI 0.87–0.99, respectively). Most other adjusted disease-specific confidence intervals included the null value. Higher body weight remained associated with opioid-priority assignment (OR = 1.06, 95% CI 1.05–1.07).

In the sensitivity analysis excluding CCI from the adjustment set, the overall pattern of disease-specific associations was broadly similar to the primary analysis (Table S7). Arthritis-related disease remained associated with slightly higher odds of NSAID-priority assignment (OR = 1.05, 95% CI 1.00–1.11), while pain-associated conditions were associated with lower odds of paracetamol-only assignment (OR = 0.94, 95% CI 0.88–0.99). Ischemic heart disease, gastritis-related disease, and respiratory disorders were associated with lower odds of NSAID-priority assignment (OR = 0.94, 95% CI 0.88–0.99; OR = 0.97, 95% CI 0.95–0.99; and OR = 0.9, 95% CI 0.83–0.99, respectively). Urinary incontinence and constipation remained associated with lower odds of opioid-priority assignment (OR = 0.93, 95% CI 0.89–0.98 and OR = 0.93, 95% CI 0.87–0.99, respectively).

## 4. Discussion

This study evaluated real-world analgesic prescribing patterns among medically complex adult outpatients aged ≥40 years and identified clinical factors associated with different hierarchical analgesic exposure groups. Analgesic prescribing at the index outpatient visit was common, with nearly three-quarters of adult outpatients aged ≥40 years receiving at least one analgesic prescription. Analgesic prescribing occurred across patients with multiple coexisting chronic conditions, particularly those with cardiovascular, metabolic, gastrointestinal, respiratory, and neuropsychiatric diseases. Arthritis-related disease was associated with slightly higher odds of NSAID-priority assignment, whereas ischemic heart disease, gastritis-related disease, and respiratory disorders were associated with lower odds of NSAID-priority assignment. Constipation and urinary incontinence were associated with lower odds of opioid-priority assignment. Concurrent prescribing of multiple analgesic classes was common, particularly among patients assigned to the opioid-priority group. The relatively high prevalence of recorded analgesic prescribing should be interpreted within the clinical context of this single-centre, multispecialty outpatient cohort. Patients were drawn from 18 clinical specialty categories, and analgesic prescribing patterns varied across specialties. These differences may partly reflect variation in the clinical characteristics and treatment needs of patients attending different services. However, because specific treatment indications and prescriber rationale were unavailable, the observed specialty differences cannot establish why individual analgesics were prescribed. Collectively, these findings illustrate the complexity of analgesic prescribing in routine outpatient practice and provide the context for the disease-specific prescribing patterns discussed below.

The principal finding of this study was that hierarchical analgesic prescribing patterns differed across patients with varying clinical and comorbidity profiles. Previous research has predominantly examined the prevalence, utilization, safety, or appropriateness of individual analgesic classes or specific patient populations [1,5,10,11]. In the present study, paracetamol, NSAIDs, and opioids were evaluated within the same outpatient cohort, with concurrent prescribing characterized separately. The hierarchical framework provided a pragmatic means of generating mutually exclusive exposure groups for comparative analysis; however, it should not be interpreted as representing the clinical sequence or rationale underlying analgesic prescribing decisions. This distinction is particularly important in adult outpatients aged ≥40 years with multimorbidity, in whom pain management commonly occurs alongside multiple chronic conditions and concurrent pharmacotherapy [3,4,8,9]. Accordingly, the contribution of this study lies primarily in characterizing how different analgesic prescribing patterns coexist with multimorbidity and clinical complexity in a large real-world outpatient population.

Another clinically important observation was that a proportion of adult outpatients aged ≥40 years with documented pain did not receive an analgesic prescription at the index outpatient visit. Similar findings have been reported in previous studies, where pain management in patients with multimorbidity was shown to be influenced by concerns regarding polypharmacy, comorbidity burden, and the potential for medication-related adverse effects rather than pain alone [1,20,21]. While this finding may raise the possibility of unmet analgesic treatment needs in some patients, alternative explanations should be considered. The present study did not capture pain severity, treatment indication, over-the-counter analgesic use, non-pharmacological pain management, patient preferences, or clinician prescribing rationale, all of which may appropriately influence the decision not to prescribe an analgesic. Accordingly, these findings should be interpreted as identifying patients who may warrant further evaluation rather than as evidence of inadequate pain management. Future longitudinal studies incorporating detailed pain assessment and treatment decision-making are needed to determine whether these prescribing patterns represent undertreatment, appropriate clinical restraint, or alternative approaches to pain management.

Another finding from the current study is that constipation and urinary incontinence were associated with modestly lower odds of opioid-priority group assignment in the final adjusted analysis. Similar inverse associations were observed in the sensitivity analyses restricted to patients with documented pain-associated conditions and in models excluding CCI (Tables S6 and S7). Both conditions may be clinically relevant when reviewing patients receiving opioid therapy, particularly because gastrointestinal effects such as opioid-induced constipation are well recognized [22,23,24]. However, the cross-sectional design prevents determination of the temporal relationship between these conditions and analgesic exposure. These findings therefore represent associations with hierarchical exposure-group assignment and should not be interpreted as evidence that the presence of constipation or urinary incontinence influenced opioid prescribing decisions or as evidence of an effect of opioid exposure on these conditions. Prospective studies incorporating treatment indication, dose, duration, symptom onset, and concomitant medications are required to clarify these relationships.

Another important finding is that ischemic heart disease and gastritis-related disease showed only small inverse associations with NSAID-priority group assignment, whereas respiratory disorders were similarly associated with lower odds of NSAID-priority assignment. Given the potential gastrointestinal, renal, cardiovascular, and respiratory considerations associated with NSAID therapy, these prescribing patterns may warrant clinical attention [7,25,26]. However, the present study did not assess treatment indication, dose, duration, contraindication, previous treatment response, or prescriber rationale and therefore cannot determine whether individual prescriptions were clinically inappropriate. Given the small magnitude of the remaining associations, they should not be interpreted as evidence of treatment appropriateness, comparative safety, or specific prescribing decisions. Future prospective studies incorporating these clinical variables are needed to determine the appropriateness and safety implications of NSAID prescribing in medically complex outpatient populations.

Adjustment for clinical specialty contributed to attenuation of several disease-specific estimates in the final models. This suggests that differences in the clinical settings in which patients were managed accounted for part of the crude associations between individual conditions and hierarchical analgesic exposure-group assignment. Clinical specialty was included as an adjustment variable to account for this potential confounding and was not evaluated as an exposure of primary interest.

Beyond individual analgesic classes, an important finding of this study was that analgesic prescribing occurred across adult outpatients aged ≥40 years with a broad range of coexisting chronic conditions, particularly cardiovascular, metabolic, gastrointestinal, respiratory, and neuropsychiatric disorders. This pattern reflects the reality that pain management in routine outpatient practice rarely occurs in isolation but is commonly undertaken within the context of complex long-term disease management. Similar observations have been reported in previous studies demonstrating that patients with multiple chronic conditions are more likely to receive analgesic therapy because chronic pain frequently coexists with other long-term illnesses and contributes substantially to treatment burden and healthcare utilization [4,9,27,28]. Our findings extend this evidence by demonstrating that these prescribing patterns span multiple analgesic classes within a large real-world outpatient population rather than being confined to a single medication class or disease group. Collectively, these results illustrate that analgesic prescribing occurs within the broader context of clinical complexity and concurrent medication use.

This study has several important strengths. First, it was based on a large real-world EHR cohort representing routine outpatient clinical practice, providing a comprehensive evaluation of analgesic prescribing across a broad spectrum of medically complex adult outpatients aged ≥40 years. Second, the inclusion of paracetamol, NSAIDs, opioids, and concurrent prescribing patterns within a single analytical framework allowed comparison of prescribing across the major analgesic classes rather than focusing on a single medication group. Third, the hierarchical exposure classification enabled the creation of mutually exclusive exposure groups for multinomial regression while preserving concurrent prescribing patterns through complementary descriptive analyses. Finally, the integration of demographic characteristics, medication burden, and multiple coexisting chronic conditions provided a broad assessment of factors associated with analgesic prescribing. Collectively, these features allowed a detailed characterization of analgesic prescribing patterns across a large and clinically diverse outpatient cohort, while the large sample size improved the precision of the estimates.

This study should be interpreted in light of several limitations. The cross-sectional design precludes determination of temporality or causality. The study was conducted at a single hospital encompassing multiple outpatient clinical specialties; therefore, the findings may not be generalizable to other healthcare systems, institutions, or outpatient populations with different specialty compositions and prescribing practices. Additionally, the study was restricted to adults aged ≥40 years; therefore, the observed prescribing patterns may not be generalizable to younger adult outpatient populations.

Information on the specific analgesic indication, pain severity and duration, analgesic dose and treatment duration, over-the-counter analgesic use, previous treatment intolerance, and prescriber rationale was unavailable. Prescription records also did not reliably distinguish newly initiated from continued therapy. Consequently, confounding by indication and residual confounding cannot be excluded, and the observed associations should be interpreted as relationships between measured clinical characteristics and prescribing patterns rather than as evidence of causal prescribing decisions, treatment appropriateness, comparative safety, or adverse drug effects.

Although analgesic prescriptions were identified at the index outpatient visit, the available data did not distinguish newly initiated treatment from continuation of existing therapy. Accordingly, the observed prevalence represents recorded prescribing at the index visit rather than the incidence of analgesic initiation or the prevalence of pain requiring treatment at that encounter. Although the principal associations were broadly preserved in a sensitivity analysis restricted to patients with documented pain-associated conditions, this analysis cannot eliminate confounding by indication because the specific indication for treatment and pain severity and duration were unavailable.

Because exposure was defined from prescriptions recorded at a single index outpatient encounter, some exposure misclassification is possible. The available data did not reliably distinguish newly initiated from continued therapy and did not capture over-the-counter analgesic use or previous prescriptions that were not recorded at the index visit.

The hierarchical classification simplified concurrent analgesic prescribing by assigning each patient to a single exposure group according to a prespecified analytical ordering. Consequently, the opioid-priority and NSAID-priority groups may include concurrent exposure to lower-priority analgesic classes, and the primary regression estimates should be interpreted as associations with hierarchical group assignment rather than exclusive exposure to an individual analgesic class. However, the principal associations were broadly preserved in non-hierarchical class-specific sensitivity analyses, providing reassurance that the main findings were not solely dependent on the imposed hierarchical ordering.

Because the primary non-user reference group may include patients without an indication for analgesic treatment, confounding by indication cannot be excluded. However, the principal associations were broadly preserved in a sensitivity analysis restricted to patients with documented pain-associated conditions, suggesting that the main findings were not solely attributable to inclusion of patients without such conditions in the non-user reference group. Nevertheless, information on the specific treatment indication, pain severity, and pain duration was unavailable, and residual confounding by indication remains possible.

In addition, because CCI was retained as a measure of overall comorbidity burden while individual clinical conditions were evaluated separately, some overlap in adjustment may remain for conditions contributing to the CCI. Accordingly, individual disease estimates should be interpreted as associations conditional on overall comorbidity burden rather than as independent causal effects. Given the large cohort, several modest effect estimates achieved statistical significance; therefore, the magnitude and clinical relevance of the associations were considered alongside statistical significance throughout the interpretation.

Although serum creatinine measurements were restricted to a ±30-day window around the index visit, renal function may have varied within this interval; additionally, the use of actual body weight in the Cockcroft–Gault equation, necessitated by the absence of height and BMI data, may have overestimated CrCl in patients with obesity.

As with all observational studies using routinely collected healthcare data, residual confounding and unmeasured clinical factors cannot be completely excluded. Despite these limitations, the large sample size and broad clinical dataset provide a useful characterization of analgesic prescribing patterns in this outpatient population and a foundation for future prospective investigations.

Analgesic prescribing should also be considered within the broader context of multimodal pain management. Chronic pain contributes substantially to disability and healthcare burden, while conservative and rehabilitation-based approaches can play an important role in managing pain and associated functional limitations [29,30]. However, non-pharmacological interventions were not captured in the present dataset, and their relationship with the observed prescribing patterns could not be evaluated.

No formal adjustment for multiple comparisons was applied across the disease-specific analyses; therefore, given the number of associations examined, the possibility of type I error cannot be excluded, and individual statistical associations should be interpreted cautiously and considered exploratory.

Our findings both support and extend the current literature on analgesic prescribing in medically complex populations. Previous studies have largely focused on the utilization, safety, and appropriateness of individual analgesic classes, providing evidence on drug-specific prescribing patterns and adverse effects. This study expands this perspective by evaluating analgesic prescribing across multiple therapeutic options rather than each analgesic class. Analgesic therapy should not be considered in isolation, because treatment selection frequently occurs within the broader context of multiple coexisting chronic conditions and concurrent pharmacotherapy. Existing recommendations emphasize consideration of patient-specific clinical characteristics, comorbidity burden, renal function, and concurrent medication use when prescribing analgesics [4,6]. In this context, the present study adds real-world evidence demonstrating that analgesic prescribing patterns differ across major analgesic classes according to patients’ underlying clinical profile [31]. Although the cross-sectional design precludes conclusions regarding treatment appropriateness or comparative safety, these findings identify associations that warrant further prospective evaluation in relation to individualized medication review and benefit–risk assessment. Prospective studies incorporating analgesic indication, pain characteristics, dose and duration, prescriber rationale, and longitudinal clinical outcomes are needed to determine the clinical significance and potential safety implications of these prescribing patterns.

## 5. Conclusions

In this large cross-sectional study of medically complex adult outpatients aged ≥40 years, analgesic prescribing was common, with NSAIDs representing the most frequently prescribed analgesic class. Hierarchical analgesic exposure patterns varied across patient clinical and comorbidity profiles. However, most disease-specific associations observed in the crude analyses were attenuated after adjustment for demographic and clinical characteristics and clinical specialty, and the remaining associations were generally modest in magnitude. The hierarchical framework provided a pragmatic approach for characterizing prescribing across major analgesic classes while accounting for concurrent exposure. Given the cross-sectional design and the absence of detailed information on treatment indication, pain severity, treatment duration, and prescribing rationale, the observed associations should not be interpreted as evidence of causal prescribing decisions or treatment appropriateness.

## Figures and Tables

**Table 1:** Demographic and clinical characteristics of the study population according to hierarchical analgesic exposure group.

Characteristics	Whole Cohort	Analgesic Non-Users	Paracetamol-Only Group	NSAID-Priority Group	Opioid-Priority Group	*p* Value	Effect Size
	n = 41,083	n = 11,700	n = 7643	n = 14,021	n = 7719		
Age, median (IQR)	52 (45–62)	52 (45–62)	52 (45–62)	52 (45–63)	52 (45–62)	0.02	ε^2^ < 0.001
Body weight, mean (SD)	75 (17)	75 (19)	74 (18)	73 (17)	80 (12)	<0.001	η^2^ = 0.021
CCI, median (IQR)	2 (1–3)	2 (1–3)	2 (1–3)	2 (1–3)	2 (1–3)	0.013	ε^2^ < 0.001
CrCl, mean (SD)	99 (42)	100 (45)	97 (43)	96 (41)	104 (38)	<0.001	η^2^ = 0.005
NPM, median (IQR)	4 (2–9)	4 (2–9)	4 (2–9)	4 (2–9)	4 (2–9)	<0.001	ε^2^ = 0.001
Gender (Male), n (%)	20,283 (49)	5778 (49)	3648 (48)	6711 (48)	4146 (54)	<0.001	V = 0.044
(Female), n (%)	20,800 (51)	5922 (51)	3995 (52)	7310 (52)	3573 (46)	<0.001	V = 0.044
Osteoporosis, n (%)	7912 (19)	2560 (22)	1525 (20)	2658 (19)	1169 (15)	<0.001	V = 0.058
Arthritis related diseases, n (%)	19,504 (48)	5688 (49)	3706 (49)	6751 (48)	3359 (44)	<0.001	V = 0.038
Pain-associated conditions, n (%)	25,257 (62)	7428 (64)	4683 (61)	8596 (61)	4550 (59)	<0.001	V = 0.032
Heart failure, n (%)	6279 (15)	1984 (17)	1164 (15)	2139 (15)	992 (13)	<0.001	V = 0.038
Hypertension, n (%)	6532 (16)	2071 (18)	1211 (16)	2199 (16)	1051 (14)	<0.001	V = 0.038
Ischemic heart diseases, n (%)	7431 (18)	2359 (20)	1372 (18)	2505 (18)	1195 (16)	<0.001	V = 0.041
Diabetes mellitus, n (%)	4672 (11)	1472 (13)	887 (12)	1575 (11)	738 (10)	<0.001	V = 0.032
Gastritis-related diseases, n (%)	13,563 (33)	4129 (35)	2560 (34)	4630 (33)	2244 (29)	<0.001	V = 0.045
Epilepsy, n (%)	2247 (6)	746 (6)	432 (6)	751 (5)	318 (4)	<0.001	V = 0.034
Depression, n (%)	1599 (4)	536 (5)	321 (4)	559 (4)	183 (2)	<0.001	V = 0.04
Urinary incontinence, n (%)	2734 (7)	830 (7)	545 (7)	918 (7)	441 (6)	<0.001	V = 0.021
Constipation, n (%)	3842 (9)	1140 (10)	746 (10)	1356 (10)	600 (8)	<0.001	V = 0.025
Respiratory disorders, n (%)	3586 (9)	1187 (10)	687 (9)	1164 (8)	548 (7)	<0.001	V = 0.038

Data are presented as mean (SD), median (IQR), or n (%), as appropriate. *p* values represent overall comparisons across the four hierarchical analgesic exposure groups and were calculated using one-way analysis of variance (ANOVA) for normally distributed continuous variables, the Kruskal–Wallis test for non-normally distributed continuous variables, and the chi-square test for categorical variables. Effect sizes are reported as η^2^ for ANOVA, ε^2^ for Kruskal–Wallis tests, and Cramer’s V for chi-square tests. IQR; interquartile range, SD; standard deviation, CCI; Chalrson comorbidity index, CrCl; creatinine clearance, NPM; number of prescribed medication, NSAID; non-steroidal anti-inflammatory drug.

## Data Availability

The datasets analyzed during the current study will be made available on reasonable request. Data will be made available for scientific purposes for researchers whose proposed use of the data has been approved by the research team.

## Supplementary Materials

The following supporting information can be downloaded at: https://www.mdpi.com/article/10.3390/jcm15208013/s1, Table S1: Comparison of Clinical Characteristics Between Male and Female Patients Prescribed Analgesics, Table S2: Comparison of Clinical Characteristics Between Older (≥65 Years) and Middle-Aged (40–64 Years) Patients Prescribed Analgesics, Table S3: Multinomial Logistic Regression Analysis of Factors Associated With Hierarchical Analgesic Exposure-Group Assignment, Table S4: Analgesic Prescribing Patterns According to Clinical Specialty at the Index Outpatient Visit, Table S5: Sensitivity Analysis of Factors Associated With Class-Specific Analgesic Prescribing Using Non-Mutually Exclusive Exposure Definitions, Table S6: Sensitivity Analysis of Factors Associated With Hierarchical Analgesic Exposure-Group Assignment Among Patients With Documented Pain-Associated Conditions, Table S7: Sensitivity Analysis of Factors Associated With Hierarchical Analgesic Exposure-Group Assignment Excluding the Chalrson Comorbidity Index from the Adjustment model.
