# Supplementary material for: Analgesic Prescribing in Medically Complex Adult Outpatients Aged ≥40 Years: A Hierarchical Analysis of Comorbidity-Associated Patterns
Source: J Clin Med. 2026 Oct 16;15(20):8013. doi: 10.3390/jcm15208013 (PMC13705678; doi:10.3390/jcm15208013)
Supplement: Supplementary file 1 [file jcm-15-08013-s001.zip › jcm-4566624-supplementary.pdf]

Analgesic Prescribing in Medically Complex Adult Outpatients Aged ≥40 Years: A Hierarchical Analysis of Comorbidity-Associated Patterns

Supplementary materials

Table S1: Comparison of clinical characteristics between male and female patients prescribed analgesics

Characteristics	Analgesic users	Male	Female	p-value
	n = 29383	n = 14505	n = 14878	
Age, mean (SD)	55.1 (12.4)	55 (12.2)	55.2 (12.6)	0.2
Body weight, mean (SD)	75 (16)	78 (16)	73 (16)	< 0.001
Charlson Comorbidity Index, median (IQR)	2 (1 – 3)	2 (1 – 3)	2 (1 – 3)	0.07
Creatinine clearance, mean (SD)	98 (41)	109 (43)	87 (35)	< 0.001
Number of prescribed medications, median (IQR)	4 (2 – 9)	4 (2 – 9)	4 (2 – 9)	0.2
Osteoporosis, n (%)	5352 (18)	2516 (17)	2836 (18)	< 0.001
Arthritis-related diseases, n (%)	13816 (47)	6703 (46)	7113 (48)	0.006
Pain- associated conditions, n (%)	17829 (61)	8655 (60)	9174 (62)	< 0.001
Heart failure, n (%)	4295 (15)	2077 (14)	2218 (15)	0.2
Hypertension, n (%)	4461 (15)	2169 (15)	2292 (15)	0.3
Ischemic heart diseases, n (%)	5072 (17)	2460 (17)	2612 (18)	0.2
Diabetes mellitus, n (%)	3200 (11)	1526 (10)	1674 (11)	0.04
Gastritis-related diseases, n (%)	9434 (32)	4531 (31)	4903 (33)	0.002
Epilepsy, n (%)	1501 (5)	700 (5)	801 (6)	0.03
Depression, n (%)	1063 (4)	473 (3)	590 (4)	0.001
Urinary incontinence, n (%)	1904 (6)	902 (6)	1002 (7)	0.07
Constipation, n (%)	2702 (9)	1322 (9)	1380 (9)	0.6
Respiratory disorders, n (%)	2399 (8)	1117 (8)	1282 (9)	0.004

SD; standard deviation; IQR; interquartile range.

Table S2: Comparison of clinical characteristics between older (≥65 years) and middle-aged (40–64 years) patients prescribed analgesics

Characteristics	Analgesic users<br>n = 29383	Older<br>n = 5918	Middle-aged<br>n = 23465	p-value
Age, mean (SD)	75 (16)	76 (18)	75 (16)	< 0.001
Body weight, mean (SD)	2 (1 – 3)	2 (1 – 3)	2 (1 – 3)	0.5
Charlson Comorbidity Index, median (IQR)	98 (41)	94 (46)	99 (40)	< 0.001
Creatinine clearance, mean (SD)	4 (2 – 9)	4 (2 – 9)	4 (2 – 9)	0.3
Number of prescribed medications, median (IQR)	5352 (18)	1433 (24)	3919 (17)	< 0.001
Osteoporosis, n (%)	13816 (47)	2343 (40)	11473 (49)	< 0.001
Arthritis-related diseases, n (%)	17829 (61)	3436 (58)	14393 (61)	< 0.001
Pain- associated conditions, n (%)	4295 (15)	1924 (33)	2371 (10)	< 0.001
Heart failure, n (%)	4461 (15)	2016 (34)	2445 (10)	< 0.001
Hypertension, n (%)	5072 (17)	2201 (37)	2871 (12)	< 0.001
Ischemic heart diseases, n (%)	3200 (11)	1372 (23)	1828 (8)	< 0.001
Diabetes mellitus, n (%)	9434 (32)	2495 (42)	6939 (30)	< 0.001
Gastritis-related diseases, n (%)	1501 (5)	462 (8)	1039 (4)	< 0.001
Epilepsy, n (%)	1063 (4)	219 (4)	844 (4)	0.7
Depression, n (%)	1904 (6)	734 (12)	1170 (5)	< 0.001
Urinary incontinence, n (%)	2702 (9)	913 (15)	1789 (8)	< 0.001
Constipation, n (%)	2399 (8)	691 (12)	1708 (7)	< 0.001

SD; standard deviation; IQR; interquartile range.

Table S3. Multinomial logistic regression analysis of factors associated with hierarchical analgesic exposure-group assignment.

Characteristic	Paracetamol-only		NSAID-priority		Opioid-priority	
	Crude (OR)	Adjusted (OR)	Crude (OR)	Adjusted (OR)	Crude (OR)	Adjusted (OR)
Age	1.00 (1.00 – 1.01)	1.00 (0.99 – 1.01)	1.003 (1.001 – 1.01)	1.002 (0.999 – 1.01)	1.00 (0.99 – 1.002)	1.00 (0.99 – 1.004)
Body weight	0.99 (0.99 – 0.99)	1.00 (1.00 – 1.01)	0.99 (0.99 – 0.99)	1.001 (0.999 – 1.003)	1.012 (1.011 – 1.014)	1.06 (1.06 – 1.07)
Creatinine Clearance	0.99 (0.99 – 0.99)	1.00 (0.99 – 1.00)	0.998 (0.997 – 0.998)	1.001 (1.00– 1.001)	1.002 (1.002 – 1.003)	1.00 (0.99 – 1.001)
Charlson Comorbidity Index	1.01 (0.99 – 1.02)	0.99 (0.97 – 1.01)	1.01 (1.003 – 1.03)	0.98 (0.97 – 1.001)	1.01 (0.99 – 1.02)	0.99 (0.97 – 1.01)
Number of prescribed medications	1.00 (0.99 – 1.01)	1.00 (0.99 – 1.01)	1.00 (0.99 – 1.01)	1.00 (0.99 – 1.01)	0.992 (0.987 – 0.998)	0.998 (0.99 – 1.004)
Sex (M)	0.94 (0.88 – 0.99)	0.99 (0.93 – 1.06)	0.94 (0.90 – 0.99)	1.03 (0.97 – 1.08)	1.19 (1.12 – 1.26)	1.03 (0.97 – 1.10)
Osteoporosis	0.89 (0.83 – 0.96)	0.97 (0.90 – 1.04)	0.84 (0.79 – 0.89)	0.96 (0.90 – 1.02)	0.64 (0.59 – 0.69)	1.01 (0.93 – 1.10)
Arthritis-related diseases	0.99 (0.94 – 1.05)	1.04 (0.98 – 1.10)	0.98 (0.94 – 1.03)	1.05 (1.00 – 1.11)	0.81 (0.77 – 0.86)	1.04 (0.97 – 1.11)
Pain-associated conditions	0.91 (0.86 – 0.97)	0.99 (0.93 – 1.06)	0.91 (0.87 – 0.96)	0.95 (0.90 – 1.00)	0.83 (0.78 – 0.88)	0.94 (0.88 – 1.00)
Heart failure	0.88 (0.81 – 0.95)	0.96 (0.89 – 1.04)	0.88 (0.83 – 0.94)	1.01 (0.95 – 1.09)	0.72 (0.67 – 0.78)	0.95 (0.86 – 1.03)
Hypertension	0.88 (0.81 – 0.95)	0.96 (0.88 – 1.03)	0.87 (0.81 – 0.92)	0.99 (0.92 – 1.06)	0.73 (0.68 – 0.79)	0.96 (0.88 – 1.05)
Ischemic heart diseases	0.87 (0.80 – 0.93)	0.95 (0.88 – 1.02)	0.86 (0.81 – 0.92)	0.97 (0.95 – 0.99)	0.73 (0.67 – 0.78)	0.96 (0.88 – 1.04)
Diabetes mellitus	0.91 (0.84 – 0.99)	0.99 (0.90 – 1.08)	0.88 (0.82 – 0.95)	1.00 (0.92 – 1.08)	0.74 (0.67 – 0.81)	0.98 (0.89 – 1.09)
Gastritis-related diseases	0.92 (0.87 – 0.98)	0.98 (0.92 – 1.05)	0.91 (0.86 – 0.95)	0.97 (0.94 – 0.99)	0.75 (0.71 – 0.80)	0.97 (0.90 – 1.03)
Epilepsy	0.88 (0.78 – 0.99)	0.95 (0.84 – 1.08)	0.83 (0.75 – 0.92)	0.95 (0.85 – 1.06)	0.63 (0.55 – 0.72)	0.88 (0.76 – 1.02)
Depression	0.91 (0.79 – 1.05)	1.02 (0.88 – 1.17)	0.87 (0.77 – 0.98)	1.02 (0.90 – 1.16)	0.51 (0.43 – 0.60)	0.85 (0.71 – 1.02)
Urinary incontinence	1.01 (0.90 – 1.13)	1.09 (0.97 – 1.22)	0.92 (0.83 – 1.01)	1.03 (0.93 – 1.14)	0.79 (0.70 – 0.89)	0.93 (0.87 – 0.99)
Constipation	1.00 (0.91 – 1.10)	1.06 (0.96 – 1.17)	0.99 (0.91 – 1.08)	1.08 (0.99 – 1.18)	0.78 (0.70 – 0.87)	0.89 (0.82 – 0.96)
Respiratory disorders	0.88 (0.79 – 0.97)	0.94 (0.85 – 1.04)	0.80 (0.74 – 0.87)	0.90 (0.83 – 0.99)	0.68 (0.61 – 0.75)	0.92 (0.83 – 1.04)

Values are presented as odds ratios (ORs) with 95% confidence intervals (CIs). The analgesic non-user group was the reference category. Models were adjusted for age, sex, body weight, Creatinine clearance, Charlson Comorbidity Index, Number of prescribed medications, and clinic specialty. Each clinical condition was evaluated separately while retaining the core adjustment variables. NSAID; non-steroidal anti-inflammatory drugs

Table S4. Analgesic prescribing patterns according to clinical specialty at the index outpatient visit

Clinical specialty	Total, n	Any analgesic, n (%)	Non-user, n (%)	Paracetamol-only, n (%)	NSAID-priority, n (%)	Opioid-priority, n (%)
Cardiology	1,583	594 (37.5)	989 (62.5)	274 (17.3)	252 (15.9)	68 (4.3)
Dental	1,581	602 (38.1)	979 (61.9)	303 (19.2)	258 (16.3)	41 (2.6)
Dermatology	562	212 (37.7)	350 (62.3)	100 (17.8)	91 (16.2)	21 (3.7)
Endocrinology	664	286 (43.1)	378 (56.9)	167 (25.2)	88 (13.3)	31 (4.7)
Otorhinolaryngology (ENT)	1,625	967 (59.5)	658 (40.5)	322 (19.8)	579 (35.6)	66 (4.1)
Gastroenterology	747	585 (78.3)	162 (21.7)	123 (16.5)	305 (40.8)	157 (21.0)
Hematology	413	305 (73.8)	108 (26.2)	72 (17.4)	159 (38.5)	74 (17.9)
Infectious Diseases	587	405 (69.0)	182 (31.0)	117 (19.9)	162 (27.6)	126 (21.5)
Internal Medicine	22,627	16,482 (72.8)	6,145 (27.2)	4,647 (20.5)	8,819 (39.0)	3,016 (13.3)
Nephrology	307	247 (80.5)	60 (19.5)	56 (18.2)	112 (36.5)	79 (25.7)
Neurology	2,531	1,989 (78.6)	542 (21.4)	420 (16.6)	827 (32.7)	742 (29.3)
Gynecology	1,009	814 (80.7)	195 (19.3)	163 (16.2)	332 (32.9)	319 (31.6)
Orthopedics	3,257	2,649 (81.3)	608 (18.7)	494 (15.2)	1,016 (31.2)	1,139 (35.0)
Physical Medicine	62	49 (79.0)	13 (21.0)	4 (6.5)	20 (32.3)	25 (40.3)
Psychiatry	571	485 (84.9)	86 (15.1)	72 (12.6)	162 (28.4)	251 (44.0)
Pulmonology	381	336 (88.2)	45 (11.8)	42 (11.0)	120 (31.5)	174 (45.7)
Urology	846	806 (95.3)	40 (4.7)	75 (8.9)	215 (25.4)	516 (61.0)
Other	1,730	1570 (90.8)	160 (9.2)	192 (11.1)	504 (29.1)	874 (50.5)
Overall	41,083	29,383 (71.5)	11,700 (28.5)	7,643 (18.6)	14,021 (34.1)	7,719 (18.8)

Analgesic exposure groups were defined hierarchically as paracetamol-only, NSAID-priority, and opioid-priority. Patients receiving opioids were assigned to the opioid-priority group regardless of concurrent NSAID or paracetamol prescribing. Patients receiving NSAIDs without opioids were assigned to the NSAID-priority group regardless of concurrent paracetamol prescribing. Percentages were calculated using the total number of patients within each clinical specialty as the denominator.

Table S5. Sensitivity analysis of factors associated with class-specific analgesic prescribing using non-mutually exclusive exposure definitions.

Characteristics	Any Paracetamol		Any NSAID		Any Opioid	
	Crude (OR)	Adjusted (OR)	Crude (OR)	Adjusted (OR)	Crude (OR)	Adjusted (OR)
Age	1.002 (1.00 – 1.003)	1.00 (0.99 – 1.003)	1.002 (1.00 – 1.003)	1.00 (0.99 – 1.00)	0.998 (0.996 – 1)	0.99 (0.99 – 1.00)
Body weight	1.002 (1.001 – 1.004)	1.003 (1.002 – 1.01)	1.001 (1.00 – 1.002)	1.00 (0.99 – 1.00)	1.02 (1.02 – 1.02)	1.01 (1.01 – 1.02)
Creatinine Clearance	1.00 (0.999 – 1.002)	1.00 (0.99 – 1.00)	1.00 (0.99 – 1.00)	1.00 (1.00 – 1.001)	1.003 (1.003 – 1.004)	1.00 (0.99 – 1.00)
Charlson Comorbidity Index	1.02 (1.01 – 1.03)	0.99 (0.98 – 1.01)	1.01 (1.002 – 1.02)	0.99 (0.98 – 1.01)	1.00 (0.99 – 1.01)	0.99 (0.98 – 1.01)
Number of prescribed medications	0.996 (0.99 – 1.00)	0.99 (0.99 – 1.00)	0.99 (0.99 – 1.00)	1.00 (0.99 – 1.00)	0.99 (0.99 – 0.99)	1.00 (0.99 – 1.00)
Sex (Male)	0.93 (0.90 – 0.97)	0.93 (0.89 – 0.97)	0.98 (0.94 – 1.02)	0.99 (0.94 – 1.03)	0.81 (0.77 – 0.85)	0.89 (0.84 – 0.94)
Osteoporosis	0.78 (0.74 – 0.82)	0.97 (0.92 – 1.02)	0.80 (0.76 – 0.84)	0.96 (0.91 – 1.01)	0.71 (0.66 – 0.75)	0.99 (0.92 – 1.06)
Arthritis-related diseases	0.90 (0.86 – 0.93)	1.02 (0.98 – 1.06)	0.93 (0.90 – 0.97)	1.03 (0.99 – 1.08)	0.82 (0.78 – 0.86)	0.97 (0.92 – 1.03)
Pain-associated conditions	0.90 (0.86 – 0.93)	0.96 (0.92 – 1.00)	0.93 (0.90 – 0.97)	0.99 (0.95 – 1.03)	0.88 (0.83 – 0.92)	0.97 (0.91 – 1.02)
Heart failure	0.80 (0.75 – 0.84)	0.92 (0.87 – 0.98)	0.88 (0.83 – 0.93)	1.00 (0.94 – 1.06)	0.78 (0.73 – 0.84)	0.92 (0.86 – 1.00)
Hypertension	0.81 (0.77 – 0.85)	0.94 (0.88 – 0.99)	0.87 (0.83 – 0.92)	0.99 (0.94 – 1.05)	0.80 (0.75 – 0.86)	0.95 (0.88 – 1.03)
Ischemic heart diseases	0.80 (0.76 – 0.84)	0.92 (0.87 – 0.97)	0.87 (0.83 – 0.91)	0.95 (0.92 – 0.99)	0.80 (0.75 – 0.85)	0.95 (0.89 – 1.02)
Diabetes mellitus	0.82 (0.77 – 0.87)	0.95 (0.89 – 1.01)	0.86 (0.81 – 0.92)	0.97 (0.91 – 1.04)	0.79 (0.73 – 0.86)	0.95 (0.87 – 1.04)
Gastritis-related diseases	0.83 (0.80 – 0.87)	0.94 (0.90 – 0.99)	0.89 (0.86 – 0.93)	0.97 (0.95 – 0.99)	0.80 (0.76 – 0.84)	0.95 (0.89 – 1.00)
Epilepsy	0.78 (0.71 – 0.85)	0.92 (0.84 – 1.01)	0.82 (0.75 – 0.89)	0.94 (0.86 – 1.03)	0.70 (0.62 – 0.79)	0.87 (0.77 – 0.99)
Depression	0.72 (0.65 – 0.79)	0.91 (0.82 – 1.01)	0.80 (0.72 – 0.89)	0.98 (0.88 – 1.09)	0.55 (0.47 – 0.64)	0.77 (0.65 – 0.90)
Urinary incontinence	0.91 (0.85 – 0.99)	1.05 (0.97 – 1.14)	0.88 (0.82 – 0.95)	0.98 (0.90 – 1.06)	0.82 (0.74 – 0.91)	0.94 (0.84 – 1.05)
Constipation	0.88 (0.82 – 0.94)	0.99 (0.93 – 1.07)	0.92 (0.89 – 0.98)	1.02 (0.95 – 1.09)	0.78 (0.72 – 0.86)	0.91 (0.85 – 0.99)
Respiratory disorders	0.76 (0.71 – 0.81)	0.89 (0.83 – 0.96)	0.80 (0.75 – 0.86)	0.91 (0.85 – 0.98)	0.76 (0.69 – 0.84)	0.96 (0.86 – 1.05)

Values are presented as odds ratios (ORs) with 95% confidence intervals (CIs). Each clinical condition was evaluated in a separate binary logistic regression model adjusted for age, sex, body weight, Creatinine clearance, Charlson Comorbidity Index, Number of prescribed medications, and clinic specialty. Exposure categories were non-mutually exclusive; therefore, an individual could contribute to more than one analgesic class. NSAID; non-steroidal anti-inflammatory drug.

Table S6. Sensitivity analysis of factors associated with hierarchical analgesic exposure-group assignment among patients with documented pain-associated conditions.

Characteristics	Paracetamol-only		NSAID-priority		Opioid-priority	
	Crude (OR)	Adjusted (OR)	Crude (OR)	Adjusted (OR)	Crude (OR)	Adjusted (OR)
Age	1.002 (1.00 – 1.005)	1.00 (0.99 – 1.004)	1.004 (1.001 – 1.006)	1.00 (0.99 – 1.01)	1.00 (0.99 – 1.003)	0.996 (0.99 – 1.00)
Body weight	0.995 (0.993 – 0.997)	1.004 (1.001 – 1.008)	0.992 (0.99 – 0.994)	1.00 (0.99 – 1.01)	1.01 (1.009 – 1.013)	1.06 (1.05 – 1.07)
Creatinine clearance	0.999 (0.998 – 0.999)	1.00 (0.99 – 1.001)	0.997 (0.997 – 0.998)	1.00 (0.999 – 1.001)	1.002 (1.001 – 1.002)	1.00 (0.998 – 1.001)
Charlson Comorbidity Index	1.02 (1.00 – 1.04)	1.01 (0.98 – 1.03)	1.02 (1.01 – 1.03)	0.99 (0.97 – 1.01)	1.01 (0.99 – 1.03)	1.01 (0.98 – 1.03)
Number of prescribed medications	0.999 (0.99 – 1.01)	1.00 (0.99 – 1.004)	1.00 (0.99 – 1.01)	0.998 (0.99 – 1.00)	0.993 (0.99 – 1.00)	0.99 (0.99 – 1.01)
Sex (Male)	0.94 (0.87 – 1.01)	1.01 (0.93 – 1.09)	0.90 (0.85 – 0.96)	0.99 (0.93 – 1.07)	1.12 (1.03 – 1.20)	0.99 (0.91 – 1.07)
Osteoporosis	0.87 (0.80 – 0.95)	0.96 (0.88 – 1.05)	0.79 (0.74 – 0.85)	0.94 (0.87 – 1.02)	0.60 (0.54 – 0.66)	1.01 (0.91 – 1.12)
Arthritis-related diseases	1.00 (0.93 – 1.08)	1.07 (0.99 – 1.15)	0.97 (0.91 – 1.04)	1.08 (1.01 – 1.15)	0.75 (0.70 – 0.81)	1.06 (0.97 – 1.15)
Heart failure	0.88 (0.80 – 0.99)	1.00 (0.90 – 1.11)	0.86 (0.79 – 0.94)	1.03 (0.94 – 1.13)	0.62 (0.55 – 0.69)	0.90 (0.79 – 1.01)
Hypertension	0.90 (0.82 – 0.99)	1.00 (0.91 – 1.11)	0.84 (0.77 – 0.91)	0.99 (0.90 – 1.08)	0.63 (0.57 – 0.71)	0.90 (0.80 – 1.02)
Ischemic heart diseases	0.90 (0.82 – 0.99)	1.00 (0.91 – 1.10)	0.85 (0.78 – 0.92)	1.00 (0.92 – 1.10)	0.63 (0.57 – 0.70)	0.91 (0.82 – 1.02)
Diabetes mellitus	0.87 (0.78 – 0.98)	0.96 (0.85 – 1.07)	0.82 (0.74 – 0.90)	0.95 (0.85 – 1.05)	0.65 (0.57 – 0.73)	0.91 (0.79 – 1.04)
Gastritis-related diseases	0.96 (0.89 – 1.04)	1.04 (0.96 – 1.12)	0.91 (0.86 – 0.97)	0.96 (0.92 – 0.99)	0.75 (0.68 – 0.80)	1.00 (0.92 – 1.10)
Epilepsy	0.86 (0.73 – 1.00)	0.94 (0.81 – 1.11)	0.84 (0.74 – 0.96)	0.99 (0.86 – 1.13)	0.59 (0.50 – 0.71)	0.86 (0.71 – 1.04)
Depression	0.97 (0.82 – 1.15)	1.10 (0.93 – 1.31)	0.91 (0.79 – 1.05)	1.11 (0.95 – 1.29)	0.46 (0.37 – 0.57)	0.83 (0.66 – 1.05)
Urinary incontinence	0.95 (0.83 – 1.07)	1.02 (0.88 – 1.17)	0.90 (0.80 – 1.02)	1.00 (0.88 – 1.13)	0.83 (0.71 – 0.96)	0.94 (0.89 – 0.99)
Constipation	0.99 (0.89 – 1.11)	1.06 (0.95 – 1.19)	0.95 (0.86 – 1.04)	1.05 (0.94 – 1.16)	0.72 (0.64 – 0.82)	0.93 (0.87 – 0.99)
Respiratory disorders	0.90 (0.80 – 1.02)	0.98 (0.87 – 1.11)	0.82 (0.74 – 0.92)	0.95 (0.85 – 1.06)	0.63 (0.55 – 0.73)	0.95 (0.82 – 1.10)

Values are presented as odds ratios (ORs) with 95% confidence intervals (CIs). The analgesic non-user group was the reference category. Models were adjusted for age, sex, body weight, Creatinine clearance, Charlson Comorbidity Index, Number of prescribed medications, and clinic specialty. Each clinical condition was evaluated separately while retaining the core adjustment variables. The analysis was restricted to patients with documented pain-associated conditions (n = 25,257). NSAID; non-steroidal anti-inflammatory drug.

Table S7. Sensitivity analysis of factors associated with hierarchical analgesic exposure-group assignment excluding the Charlson Comorbidity Index from the adjustment model.

Characteristics	Paracetamol-only		NSAID-priority		Opioid-priority	
	Crude (OR)	Adjusted (OR)	Crude (OR)	Adjusted (OR)	Crude (OR)	Adjusted (OR)
Age	1.00 (1.00 – 1.01)	1.00 (0.99 – 1.00)	1.003 (1.001 – 1.005)	1.00 (0.99 – 1.00)	1.00 (0.998 – 1.002)	1.00 (0.99 – 1.00)
Body weight	0.995 (0.993 – 0.997)	1.00 (1.00 – 1.01)	0.992 (0.991 – 0.994)	1.00 (0.99 – 1.00)	1.012 (1.011 – 1.014)	1.06 (1.06 – 1.07)
Creatinine clearance	0.999 (0.998 – 0.999)	1.00 (0.99 – 1.00)	0.998 (0.997 – 0.998)	1.00 (1.00 – 1.00)	1.002 (1.002 – 1.003)	1.00 (0.99 – 1.00)
Number of prescribed medications	1.00 (0.99 – 1.01)	0.99 (0.99 – 1.01)	1.00 (0.99 – 1.01)	1.00 (0.99 – 1.01)	0.99 (0.987 – 0.998)	1.00 (0.99 – 1.00)
Sex (Male)	0.94 (0.88 – 0.99)	0.99 (0.94 – 1.06)	0.94 (0.90 – 0.99)	1.03 (0.98 – 1.09)	1.19 (1.12 – 1.26)	1.03 (0.97 – 1.10)
Osteoporosis	0.89 (0.83 – 0.96)	0.97 (0.90 – 1.04)	0.84 (0.79 – 0.89)	0.96 (0.90 – 1.02)	0.64 (0.59 – 0.69)	1.01 (0.93 – 1.10)
Arthritis-related diseases	1.00 (0.94 – 1.05)	1.04 (0.98 – 1.10)	0.98 (0.94 – 1.03)	1.05 (1.00 – 1.11)	0.81 (0.77 – 0.86)	1.04 (0.98 – 1.11)
Pain-associated conditions	0.91 (0.86 – 0.97)	0.94 (0.88 – 0.99)	0.91 (0.87 – 0.96)	0.95 (0.90 – 1.00)	0.83 (0.78 – 0.88)	0.94 (0.88 – 1.00)
Heart failure	0.88 (0.81 – 0.95)	0.96 (0.89 – 1.04)	0.88 (0.83 – 0.94)	1.01 (0.95 – 1.09)	0.72 (0.67 – 0.78)	0.95 (0.86 – 1.03)
Hypertension	0.88 (0.81 – 0.95)	0.95 (0.88 – 1.03)	0.87 (0.81 – 0.92)	0.99 (0.92 – 1.06)	0.73 (0.68 – 0.79)	0.96 (0.88 – 1.05)
Ischemic heart diseases	0.87 (0.81 – 0.93)	0.95 (0.88 – 1.02)	0.86 (0.81 – 0.92)	0.92 (0.88 – 0.96)	0.73 (0.67 – 0.78)	0.96 (0.88 – 1.04)
Diabetes mellitus	0.91 (0.84 – 0.99)	0.99 (0.90 – 1.08)	0.88 (0.82 – 0.95)	1.00 (0.92 – 1.08)	0.74 (0.67 – 0.81)	0.98 (0.89 – 1.09)
Gastritis-related diseases	0.92 (0.87 – 0.98)	0.98 (0.92 – 1.05)	0.90 (0.86 – 0.95)	0.97 (0.95 – 0.99)	0.75 (0.71 – 0.80)	0.97 (0.90 – 1.03)
Epilepsy	0.88 (0.78 – 0.99)	0.95 (0.84 – 1.08)	0.83 (0.75 – 0.93)	0.95 (0.85 – 1.06)	0.63 (0.55 – 0.72)	0.88 (0.76 – 1.02)
Depression	0.91 (0.80 – 1.05)	1.02 (0.88 – 1.17)	0.87 (0.77 – 0.98)	1.02 (0.90 – 1.16)	0.51 (0.43 – 0.60)	0.85 (0.71 – 1.02)
Urinary incontinence	1.00 (0.90 – 1.13)	1.09 (0.97 – 1.22)	0.92 (0.83 – 1.01)	1.03 (0.93 – 1.14)	0.79 (0.70 – 0.89)	0.93 (0.89 – 0.98)
Constipation	1.00 (0.91 – 1.10)	1.06 (0.96 – 1.27)	0.99 (0.91 – 1.08)	1.08 (0.99 – 1.18)	0.78 (0.70 – 0.87)	0.92 (0.87 – 0.99)
Respiratory disorders	0.88 (0.79 – 0.97)	0.94 (0.85 – 1.04)	0.80 (0.74 – 0.87)	0.90 (0.83 – 0.99)	0.68 (0.61 – 0.75)	0.93 (0.83 – 1.04)

Values are presented as odds ratios (ORs) with 95% confidence intervals (CIs). The analgesic non-user group was the reference category. Models were adjusted for age, sex, body weight, Creatinine clearance, Number of prescribed medications, and clinic specialty. Each clinical condition was evaluated separately while retaining the core adjustment variables. NSAID; non-steroidal anti-inflammatory drug.
